# High-temperature martensitic transformation of CuNiHfTiZr high- entropy alloys

**DOI:** 10.1038/s41598-019-55762-y

**Published:** 2019-12-20

**Authors:** Shan-Hsiu Chang, Po-Ting Lin, Che-Wei Tsai

**Affiliations:** 1 Department of Materials Science and Engineering, National Tsing Hua University, Hsinchu, 30013 Taiwan, ROC; 2 High Entropy Materials Center, National Tsing Hua University, Hsinchu, 30013 Taiwan, ROC

**Keywords:** Metals and alloys, Design, synthesis and processing

## Abstract

One of the major challenges of near-equiatomic NiTi shape memory alloys is their limitation for high-temperature applications. To overcome this barrier, researchers have tried to enhance the transformation temperatures by addition of alloying elements or even by introducing the concept of high-entropy alloys (HEAs). In this study, the CuNiHfTiZr HEAs were developed for high-temperature shape memory effect. Based on their solubility and electron configurations, the alloying elements are divided into two groups, (CuNi)_50_ and (HfTiZr)_50_. The content of Cu in (CuNi)_50_ is modulated to investigate the influences of Cu on martensitic transformation of the HEAs by studying structural evolution and transformation behavior. The results of x-ray diffraction and thermal expansion tests revealed that Cu_15_Ni_35_Hf_16.67_Ti_16.67_Zr_16.67_ possesses high transformation temperature, narrow hysteresis temperature loops, and good dimensional stability within this HEA system.

## Introduction

NiTi shape memory alloys (SMAs) are renowned for their good ductility, corrosion resistance, biocompatibility, damping behavior, and workability^1–5^. However, the applications of near-equiatomic NiTi alloys at high temperatures are limited due to the relatively low transformation temperatures (TTs) which are below 100 °C^6,7^. Given the great potential of high-temperature shape-memory applications, many researchers have attempted to increase the TTs by adding alloying elements, such as Hf, Zr, Pd, and Pt, into NiTi-based alloys. Several studies suggested that Hf, Zr, and Nb have better solubility with and similar electron configuration to Ti in Nitonol, whereas Co and Cu have better solubility with and similar electron configuration to Ni in Nitinol^6–9^. This can serve as an important concept for design of SMAs.

Recently, the novelty and exceptional properties of high-entropy alloys (HEAs) have led to extensive research and study. HEAs were proposed and defined by Yeh *et al*.^10^ as alloys that contain at least five metallic elements, each with 5–35 atomic percent. The four core effects of HEAs include high-entropy effect, large lattice distortion, sluggish diffusion, and cocktail effect^10,11^. Interestingly, Firstov *et al*.^12^ first came up with an idea that combined high-temperature shape memory alloys (HTSMAs) with high-entropy alloys (HEAs), and successfully developed the CoCuNiHfTiZr high-entropy SMA system, which was allegedly able to perform martensitic transformation with a TT over 100 °C. Among the four alloy compositions they developed, i.e., Ti_16.667_Zr_16.667_Hf_16.667_Co_25_Cu_25_, Ti_16.667_Zr_16.667_Hf_16.667_Ni_25_Cu_25_, Ti_16.667_Zr_16.667_Hf_16.667_Co_25_Ni_25_, and Ti_16.667_Zr_16.667_Hf_16.667_Co_16.667_Ni_16.667_Cu_16.667_, only Ti_16.667_Zr_16.667_Hf_16.667_Ni_25_Cu_25_ shows martensitic transformation from B2 austenite to B19’ martensite. Following this research finding, they further discovered that varying Co, Ni, and Cu content in this alloy system leads to different shape memory properties and TTs. According to their experimental findings, Co_10_Cu_15_Ni_25_Hf_16.67_Ti_16.67_Zr_16.67_ HEA exhibited higher values of TT and recovered martensitic strain. However, our experiments that tested the reproducibility of the Co_10_Cu_15_Ni_25_Hf_16.67_Ti_16.67_Zr_16.67_ HEA suggested that its martensitic TT may not be as high as previously reported (experimental proofs are provided in the supplemental file).

To provide physical principles for the development of industrial high-temperature SMAs, Firstov *et al*. made a comprehensive and thorough investigation into the electronic and crystal structures of the B2 intermetallic compounds of the AB type (A = Ti, Zr, Hf; B = Co, Ni, Cu), i.e., ZrCo, ZrNi, and ZrCu binary equiatomic intermetallic compounds^13^. They discussed the effects of several factors on shape memory behavior, including different crystal structures with different degree of symmetry, the packing density that leads to different vibrational entropy and stability of martensite, and the interactions of 3d and 4d electrons of the atoms. In addition to the study on binary intermetallic compounds, they discussed the different stability of B19’ martensite and B2 austenite in TiZrHfCoNiCu high-entropy SMA^14^, and further explained the effect of Co addition, which replaced Cu, Ni, or both, with the ab-initio modeling of the alloy’s crystal and electronic structure. Based on the theoretical modeling and experimental results mentioned above, it can be concluded that the martensitic transformation of either binary intermetallics or TiZrHfCoNiCu high-entropy SMA is mainly dominated by the competing A-B and B-B interactions, which decide the total energy and the stability of the martensite or austenite during changing of temperature^13,14^. All these findings provide important insights into the design and development of high-temperature SMAs.

Apart from the (CoCuNi)(HfTiZr) system, in a very recent work, Ni-Ti based high-entropy SMAs (Ni,Pd)_50_(Ti,Hf,Zr)_50_ with austenite finish temperature beyond 700 °C were designed with the shape memory behavior tested by Canadinc *et al*.^15^ They speculated that the enhanced configurational entropy of the alloys by multi-element alloy design is responsible for the significantly increased TTs and recovered strain at elevated temperatures. Despite the promising shape memory properties of the (Ni,Pd)_50_(Ti,Hf,Zr)_50_ alloys, the high cost arising from large proportion of Pd addition is another critical issue to deal with.

In this research, Co was eliminated from the CoCuNiHfTiZr system in the first place. In other words, the alloy system discussed in this research is CuNiHfTiZr. According to several research findings^11,16–18^, the addition of Co significantly decreases martensitic TTs, which implied that Co is a strong stabilizer of B2 austenite, the stable phase at higher temperatures^14^. Therefore, with the focus on development of SMAs with high martensitic TTs, Co was removed from the alloy system even though current research findings imply that Co can help improve yield strength^16^.

Despite the inspiring findings of high-temperature shape memory effect in HEAs, the alloy systems developed previously still require considerable improvement. This research aims not only to understand the influence of Cu addition on martensitic transformation of the CuNiHfTiZr HEAs, but also to provide insights into the development of high-entropy, high-temperature shape memory alloys.

## Results and Discussion

In this alloy system, four compositions, Cu_x_Ni_50-x_Hf_16.67_Ti_16.67_Zr_16.67_, (x = 0, 5, 15, 25), were developed with varied Cu content to determine the influence of Cu. The naming of the four alloy compositions are listed as below:

Ni_50_Hf_16.67_Ti_16.67_Zr_16.67_: Cu0

Cu_5_Ni_45_Hf_16.67_Ti_16.67_Zr_16.67_: Cu5

Cu_15_Ni_35_Hf_16.67_Ti_16.67_Zr_16.67_: Cu15

Cu_25_Ni_25_Hf_16.67_Ti_16.67_Zr_16.67_: Cu25

Cu and Ni are independent variables in the compositions, because Cu and Ni have excellent solubility and similar electron configurations. On the other hand, Hf, Ti and Zr are control variables, and were set to be equiatomic for two reasons. One is that Hf and Zr have similar electron configuration and good solubility to Ti^18^. The other reason is to comply with the definition of high-entropy alloys.

**Figure 1:**
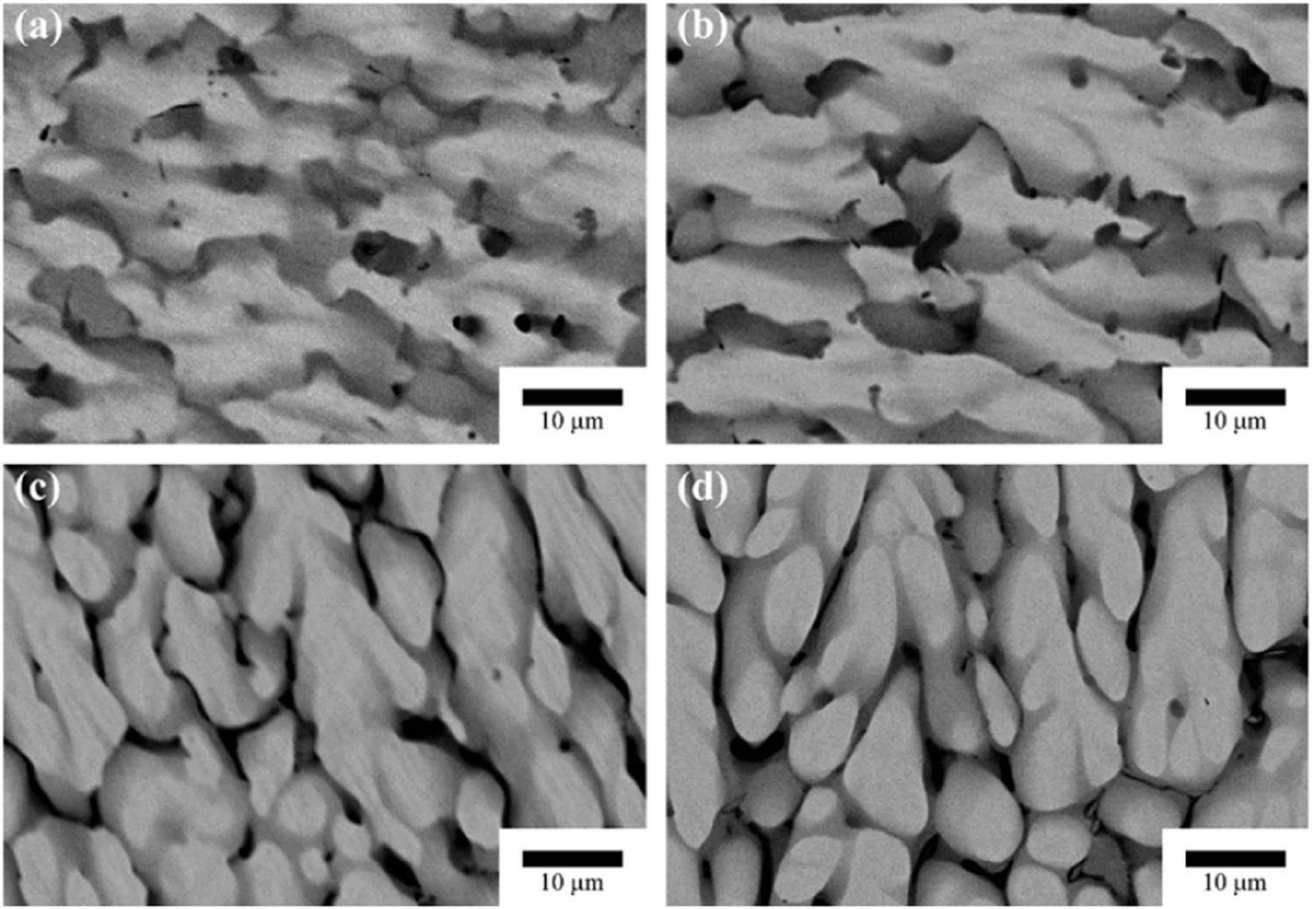
Microstructure (backscattered electrons images) of as-cast ingots: (**a**) Cu0, (**b**) Cu5, (**c)** Cu15, and (**d**) Cu25.

Four alloy compositions, Cu0, Cu5, Cu15, and Cu25, were fabricated with as-cast state. Figure 1 shows the microstructures (backscattered electrons images) of all the as-cast specimens. Due to the difference in melting points of constituent elements, the dendritic and interdendritic structures are clearly observed. The dendrite (white) of Cu0 is rich in Hf, and the interdendrite (black) is slightly enriched with Zr comparing to the white phase. The specimens with Cu addition have similar microstructure but different compositions in dendrite and interdendrite (Table 1). All the black phases are rich in Cu and Zr, and the white phases are rich in Hf and have more Ni content than the black phases.

**Table 1:** Compositional (EDS) results of Cu0 and other specimens with Cu addition.

Specimen	D/ ID	Cu (at.%)	Ni (at.%)	Hf (at.%)	Ti (at.%)	Zr (at.%)
Cu0	Dendrite	—	49.3	18.6	15.5	17.6
	Interdendrite	—	49.0	13.5	18.5	18.9
Cu5	Dendrite	4.5	45.1	18.8	15.2	16.5
	Interdendrite	6.5	42.3	13.7	19.0	18.6
Cu15	Dendrite	12.8	37.0	19.2	14.7	16.3
	Interdendrite	20.0	29.0	10.6	20.0	20.4
Cu25	Dendrite	21.6	28.4	18.8	14.7	16.6
	Interdendrite	26.5	21.2	14.8	18.1	19.3

**Figure 2:**
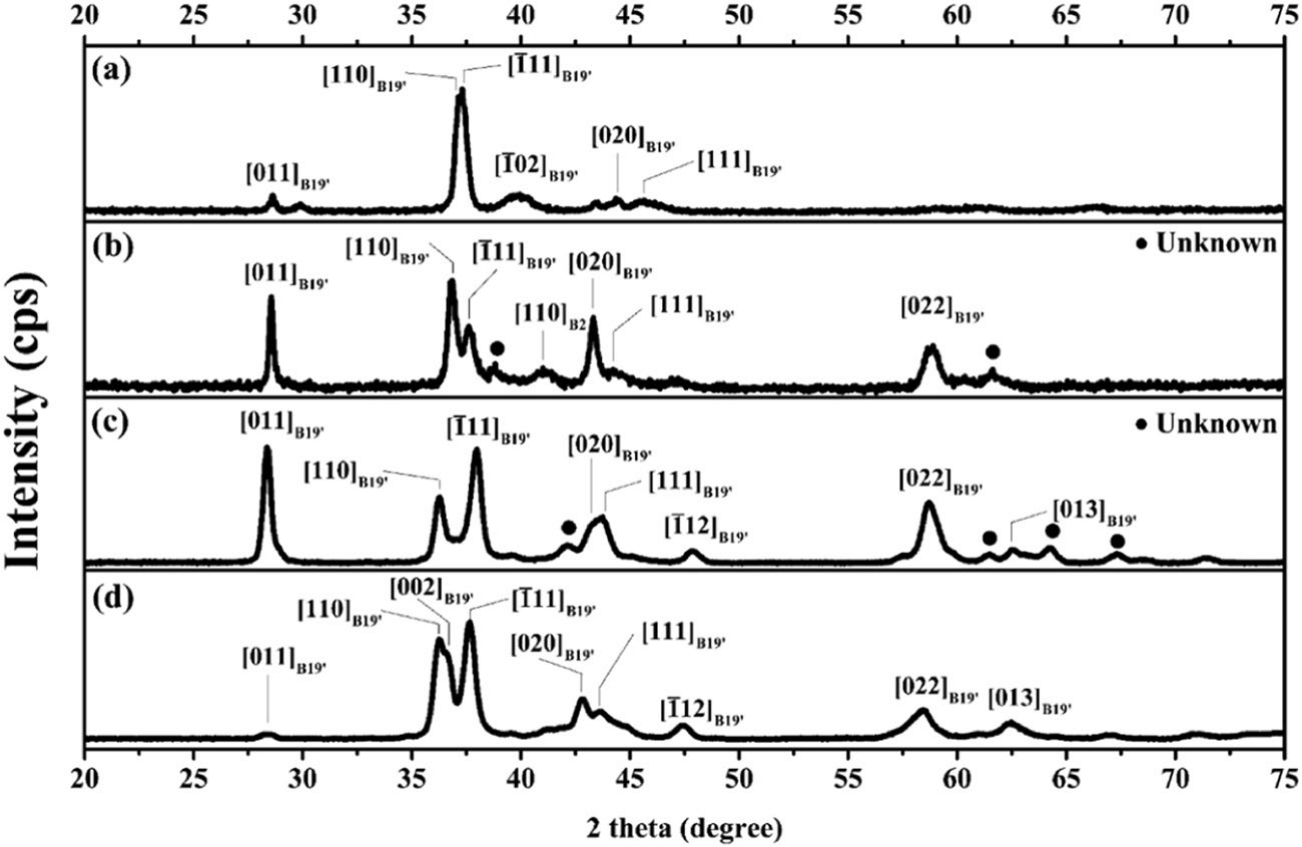
X-ray diffraction patterns at RT of (**a**) Cu0, (**b**) Cu5, **(c**) Cu15, and (**d**) Cu25.

**Figure 3:**
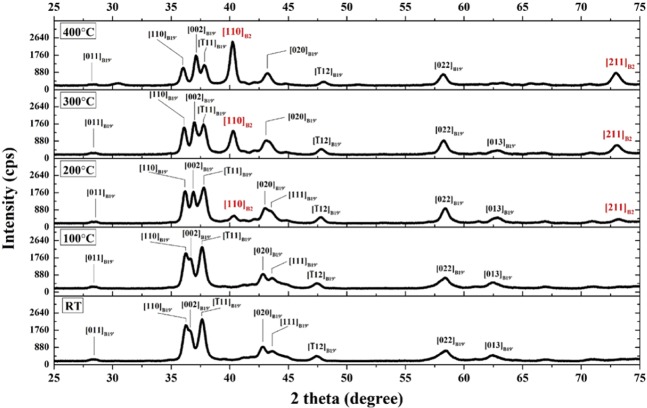
High-temperature X-ray diffraction patterns of Cu25 at RT, 100 °C, 200 °C, 300 °C, and 400 °C.

The XRD results (Fig. 2) reveal that the crystal structures of all specimens are B19’ martensite at RT with a monoclinic structure, and they are of P2_1_/m space group. Table 2 presents the lattice constants and volume of the unit cells determined with the formula $V=a\times b\times c\times {\sin }\beta$ using Maud software after Rietveld refinement. The volume of the unit cells increases with increasing content of Cu because the atomic radius of Cu (1.278 Å) is larger than that of Ni (1.246 Å). Since Cu and Ni have excellent solubility and similar electron configuration, adding Cu resulted in atom replacement of Ni with Cu, which subsequently results in larger volume of the unit cell. Figure 3 shows the XRD patterns of Cu25 alloy at RT and elevated temperatures (i.e., 100 °C, 200 °C, 300 °C and 400 °C). The values on the y-axis are specifically highlighted for comparison of peak intensity. A [110]_B2_ peak was discovered at approximately 40.2° in the 200 °C, 300 °C, and 400 °C patterns, and the intensity of this [110]_B2_ peak increased with increasing temperature, which demonstrates that this alloy system undergoes martensitic transformation between B2 and B19′ structures.

**Table 2:** Lattice constants, volume of the unit cells, and volume per atoms of Cu0 and other specimens with Cu addition (Data of Ti_16.667_Zr_16.667_Hf_16.667_Cu_25_Ni_25_, same composition as Cu25, published by Firstov *et al*.^12^ are included).

Specimen	a (Å)	b (Å)	c (Å)	β (°)	Volume (Å^3^)	Volume per Atom (Å^3^)
Cu0	3.146	4.086	5.061	107.06	62.200	15.550
Cu5	3.167	4.110	5.054	104.95	63.545	15.886
Cu15	3.159	4.118	4.947	102.75	63.922	15.981
Cu25 (B19′)	3.172	4.138	5.007	102.65	64.131	16.033
Cu25 (B2)	3.166	—	—	—	31.735	15.867
Ti_16.667_Zr_16.667_Hf_16.667_Cu_25_Ni_25_ (B19′)^12^	3.155	4.167	4.967	100.95	64.128	16.032
Ti_16.667_Zr_16.667_Hf_16.667_Cu_25_Ni_25_ (B2)^12^	3.167	—	—	—	31.783	15.892

**Figure 4:**
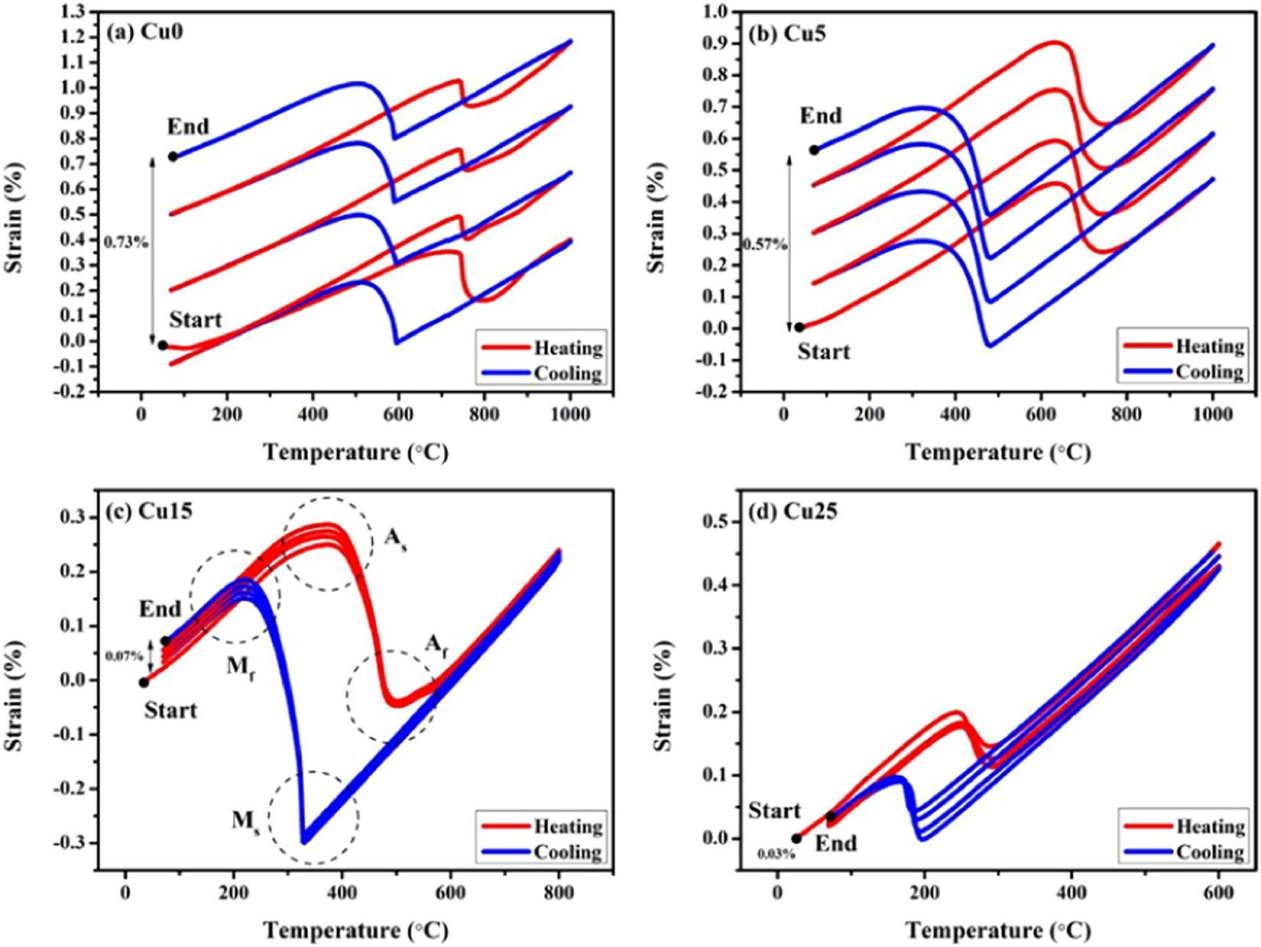
Martensitic transformation behavior of (**a**) Cu0, (**b**) Cu5, (**c**) Cu15, and (**d**) Cu25 measured by DIL during heating (red lines) and cooling (blue lines). For TTs details, please refer to Table 3.

Figure 4 shows the thermal cycling test (dilatometry) results of the four compositions and demonstrates that the CuNiHfTiZr HEA system shows martensitic transformation. During heating, the drastic drop of the strain represents the beginning of the phase transformation from martensite to austenite because martensite owns larger volume than austenite in this alloy system. When the alloys underwent cooling, the significant increase of strain indicates that martensitic transformation occurred, and austenite was transformed back into martensite. By combining the results of Table 2, Figs. 3 and 4(d), the strain drop in Fig. 4(d)can be directly related to the reverse martensitic transformation as shown in the changing XRD pattern of Fig. 3, in which the Cu25 alloy underwent phase transformation from B19′ structure with a 16.033 Å^3^ volume per atom to B2 structure with a 15.867 Å^3^ volume per atom and had a volume contraction from 0.18% to 0.11% of the strain (Fig. 4(d)). Also, it should be noted that the XRD results of Cu25 at 400 °C (Fig. 3) suggest the presence of B19′ martensite although the results of dilatometry (Fig. 4(d)) indicate the completion of inverse martensitic transformation at 400 °C. This discrepancy can be attributed to the difference in heating rate during the two analyses and the uncertain properties of the as-cast samples. The heating rate of dilatometry was 5 °C/min, while the heating rate of high-temperature XRD was 10 °C / min: the higher heating rate in the high-temperature XRD test may result in the signal of B19′ martensite whose transformation into B2 austenite is not yet complete. On the other hand, because the samples tested in this study were of as-cast state, the inhomogeneity of the alloy samples can possibly lead to some defects, which then hinder the reverse martensitic transformation and cause the formation of retained martensite that cannot successfully transform into austenite even when the A_f_ temperature is reached.

In addition to the observation of martensitic transformation, it was found that addition of Cu is conducive to improvement of dimensional stability as Cu15 and Cu25 have much higher dimensional stability comparing to Cu0 and Cu5. It should also be noted that an irreversible plastic strain formed after every heating and cooling cycle. Without the addition of Cu, the irreversible strain of Cu0 after 4 cycles was 0.73%. However, with increasing content of Cu, the irreversible strain significantly dropped to 0.57%, 0.07%, and 0.03% in Cu5, Cu15, and Cu25 alloys, respectively. These results suggested that Cu addition can effectively enhance dimensional stability and suppress the formation of irreversible strain in the CuNiHfTiZr HEA system. The underlying mechanism of this phenomenon is yet unknown, but it is speculated that the addition of Cu, although decreases TTs, can effectively increase the stability of the B19’ martensite by forming strong d-d electron interactions between Cu atoms and thus enhance dimensional stability during phase transformation.

The TTs values of the four alloy compositions in each of the four cycles are listed in Table 3. The starting and finishing temperatures of the martensitic transformation are denoted as M_s_ and M_f_. Likewise, the starting and finishing temperatures of the reverse (austenitic) transformation are denoted as A_s_ and A_f._ All TTs remained quite stable during cyclic tests. From Table 3, we can tell that Cu0 and Cu5 have higher TTs, yet their hysteresis (A_f_ - M_s_) are wider. This trend reveals that with a rather minor addition of Cu, the alloy would not be favorable to thermoelastic martensitic transformation due to wide hysteresis loops. With the Cu addition of 5, 15, and 25 atomic percent, the temperature of A_s_ in the second cycle decreased by 108 °C, 374 °C, and 495 °C, respectively, from 742 °C of Cu0. By simple calculations, the effect of Cu addition on TTs is derived to be a − 20 °C per atomic percent of Cu addition. Although adding Cu decreases the TTs, it also narrows the temperature hysteresis loops, which is in favor of thermoelastic transformation^6^.

**Table 3:** TTs of the four alloy compositions in each cycle.

(°C)	M_s_	M_f_	A_s_	A_f_	Hysteresis
**Cu0**					
Cycle 1	591	512	719	795	204
Cycle 2	591	510	742	760	169
Cycle 3	589	504	742	762	173
Cycle 4	587	506	741	771	184
**Cu5**					
Cycle 1	482	328	633	743	261
Cycle 2	481	324	634	747	266
Cycle 3	481	320	633	748	267
Cycle 4	481	325	632	750	269
**Cu15**					
Cycle 1	328	219	375	501	173
Cycle 2	328	221	368	504	176
Cycle 3	328	222	373	503	175
Cycle 4	328	220	372	503	175
**Cu25**					
Cycle 1	197	165	243	289	92
Cycle 2	194	166	247	293	99
Cycle 3	190	164	249	293	103
Cycle 4	185	161	251	290	105

Considering the dimensional stability and martensitic TTs, it is concluded that the Cu15 alloy exhibits the most promising properties for the development of high-temperature shape memory effect among the four alloy compositions and is therefore the best candidate for further research.

## Conclusions

In this research, the Cu0, Cu5, Cu15, and Cu25 alloys were developed from the CuNiHfTiZr high-entropy alloy system. The conclusions are drawn as follows:

- The as-cast CuNiHfTiZr high-entropy alloys consist of dendrites and interdendrites. In Cu0, the dendrite is rich in Hf, and the interdendrite is slightly rich in Zr. In Cu5, Cu15, and Cu25, the dendrite is rich in Hf and Ni, while the interdendrite is rich in Cu and Zr.
- The x-ray diffraction results demonstrate that Cu0, Cu5, Cu15, and Cu25 all belong to B19’ structure at RT after casting. During the rise of temperature, the crystal structure of the Cu25 alloy transforms into B2 austenite.
- Cu addition to the CuNiHfTiZr system effectively increases the volume of a unit cell from 62.200 Å^3^ of Cu0 to 64.131 Å^3^ of Cu25.
- Thermal cycling tests reveals the martensitic transformation of all four alloys, and it is found that Cu addition from 0 to 25 at.% leads to a drop in martensitic TTs from above 750 °C to less than 300 °C in terms of A_f_ temperatures. The effect of Cu addition on martensitic TTs in this alloy system is −20 °C/at.%.
- Cu addition to the CuNiHfTiZr system narrows the hysteresis loops and increases the dimensional stability, i.e., decreases irreversible strain, during thermal cycling tests.
- Among the four alloy compositions, Cu_15_Ni_35_Hf_16.67_Ti_16.67_Zr_16.67_ (Cu15) exhibits the best properties for the development of high-temperature shape memory effect and is therefore worthy of further study.
- This study only shows that CuNiHfTiZr HEAs can perform martensitic transformation at high temperatures (i.e., 200–800 °C). Further research and experiments in addition to dilatometry must be conducted to confirm high-temperature shape memory effect of the CuNiHfTiZr HEA system.
- Further study on the CuNiHfTiZr HEAs requires the investigation into the effects of different thermal/mechanical treatments given that all alloys in this study are of as-cast state.

## Methods

All alloy ingots were prepared by vacuum arc-melting, in which high-purity raw materials were mixed, turned over, and remelted at least five times to ensure compositional homogeneity. X-ray diffraction (XRD) analysis was conducted using a Bruker D2 Phaser to examine the crystal structure of the as-cast samples at RT. Transformation behavior and crystal structure at elevated temperatures were examined by *in situ* high-temperature XRD (Bruker D8 Discover) analysis, in which the heating rate was 10 °C/min. The as-cast ingots were cut into cylindrical samples for thermal analysis (dilatometry) with a diameter of 3.6 mm and a height of 6 mm by wire electrical discharge machining. NETZSCH DIL 402E Select dilatometer was used to measure the quantity of recovery and to determine the martensitic TTs. All samples underwent four cycles (i.e., four heating and cooling processes). The heating and cooling rates were both 5 °C/min, and a load of 0.2 N was applied to make sure the push rod remains in contact with samples during measurement, which would not affect the observation of TTs. Microstructural observations were conducted with a JEOL JSM-IT100 scanning electron microscope.

## Supplementary information

High-temperature martensitic transformation of CuNiHfTiZr high-entropy alloys

## Data Availability

The data supporting the findings in this study are available within the paper. Any further information or clarification is available from the corresponding author upon reasonable request.
