# Supplementary material for: High-temperature martensitic transformation of CuNiHfTiZr high- entropy alloys
Source: Sci Rep. 2019 Dec 20;9:19598. doi: 10.1038/s41598-019-55762-y (PMC6925220; doi:10.1038/s41598-019-55762-y)
Supplement: Supplementary file 1 — High-temperature martensitic transformation of CuNiHfTiZr high-entropy alloys [file 41598_2019_55762_MOESM1_ESM.docx]

High-temperature martensitic transformation of CuNiHfTiZr high- entropy alloys

Shan-Hsiu Chang^a^, Po-Ting Lin^a,b^, and Che-Wei Tsai^a,b*^

^a^Department of Materials Science and Engineering, National Tsing Hua University, Hsinchu 30013, Taiwan, ROC

^b^High Entropy Materials Center, National Tsing Hua University, Hsinchu 30013, Taiwan, ROC

**Supplemental Materials**





**Figure S1** The differential scanning calorimetry result of the Ti_16.667_Zr_16.667_Hf_16.667_Co_10_Ni_25_Cu_15_ high-entropy shape memory alloy.





**Figure S2** The dilatometry result of the Ti_16.667_Zr_16.667_Hf_16.667_Co_10_Ni_25_Cu_15_ high-entropy shape memory alloy.

**Table S1** The martensitic transformation temperatures of the Ti_16.667_Zr_16.667_Hf_16.667_Co_10_Ni_25_Cu_15_ high-entropy shape memory alloy.

| Unit: °C | **M_s_** | **M_f_** | **A_s_** | **A_f_** |
| --- | --- | --- | --- | --- |
| **DSC** | 106 | -28 | -28 | 146 |
| **DIL** | 84 | - | 65 | 145 |
